# The alcohol industry’s involvement with road safety NGOs

**DOI:** 10.1186/s12992-022-00813-9

**Published:** 2022-02-15

**Authors:** Ivy Stein, Abdulgafoor M. Bachani, Connie Hoe

**Affiliations:** 1 International Injury Research Unit, Johns Hopkins Bloomberg School of Public Health, Baltimore, MD USA; 2 Heidelberg Institute for Global Health, Faculty of Medicine and University Hospital, Heidelberg University, Im Neuenheimer Feld 130.3, 69120 Heidelberg, Germany

**Keywords:** Alcohol industry, Road safety, Industry interference, Drink-driving

## Abstract

**Background:**

Road crashes are a major cause of death among all age groups and the leading cause of death among persons 5–29 years, according to the World Health Organization. One key risk factor is drink-driving. While the world’s leading beer, wine, and spirit producers have pledged to combat drink-driving, there is increasing evidence showing the alcohol industry’s promotion of solutions which minimally impact sales. One strategy is forming partnerships with road safety non-governmental organizations (NGOs). Given this, the primary objective of this study is to understand the extent to which the alcohol industry is involved with road safety NGOs around the world.

**Methods:**

A desk review from July 2020 to March 2021 was conducted to assess the alcohol industry’s involvement with various road safety NGOs (*n* = 256) in 92 countries. Financial documents press releases, annual reports, social media platforms, and other resources were analyzed to uncover relationships between the alcohol industry and NGOs.

**Results:**

Out of 256 NGOs, *n* = 11 (4%) showed direct ties to the alcohol industry, and *n* = 3 (1%) showed indirect ties. NGOs involved with the alcohol industry were found in five continents and *n* = 8 of the 11 NGOs (73%) partnered with transnational alcohol manufacturers. Interventions supported by these partnerships were primarily mass media campaigns, free-ride and ride-sharing campaigns, and drink-driving educational events where alcoholic or zero-percent alcoholic beverages were sold or provided. These interventions are largely inconsistent with evidence-based best practice recommendations. Relationships between the alcohol industry and road safety NGOs lacked public transparency on key details such as terms of partnerships and funding amount and terms.

**Conclusions:**

The study showed a clear effort on behalf of the alcohol industry to partner with road safety NGOs around the world. Findings underscore the need for the road safety community to generate consensus on involvement of the alcohol industry and suggest the need for more transparency on details of partnerships involving road safety. Findings also highlight the importance of local and national government support of road safety initiatives and road safety NGOs to avoid dependence on controversial funding from the alcohol industry.

## Background

Globally, road crashes are a major cause of death among all age groups and the leading cause of death among persons aged 5–29 years [1]. Approximately 1.35 million people die on the road annually, while another 20 to 50 million are injured [1]. Over 90% of these crashes occur in low- and middle-income countries, according to the World Health Organization (WHO) [1]. It is estimated that anywhere from 5 to 35% of road traffic deaths in the world are attributable to alcohol [1]. Alcohol is a depressant that slows activity of the brain and the rest of the central nervous system, leading to decreased ability to safely operate a vehicle [2].

Many large players within the alcohol industry, namely the world’s leading beer, wine, and spirit producers, admit drink-driving is a problem they must confront [3] and have placed the issue at the forefront of their corporate social responsibility (CSR) agendas [4].

However, while interventions are employed by the alcohol industry to reduce the prevalence of drink-driving around the world, growing evidence suggests that the majority of interventions supported by the industry have limited to no public health evidence base and will have minimal effect on sales [5, 6]. The financial motives of the industry are at strong odds with public health aims to reduce harm caused by alcohol – while one requires an increase in alcohol sales and consumption, the other necessitates a reduction [7]. Additionally, Babor et al. [8] found the alcohol industry to be concentrated globally into a small number of transnational corporations, allowing for greater policy influence and market expansion, as well as the enhanced use of CSR tactics which create a favorable regulatory environment.

Public health experts explain that road safety interventions used by the alcohol industry are designed to boost brand reputation and serve the interests of alcohol industry actors, rather than directly and effectively address issues such as drink-driving [9]. Studies also show that the alcohol industry’s CSR approaches positively influence consumers’ perceptions and raise the market value for the industry’s goods [10, 11]. Public health advocates believe the industry’s assertions of responsibility through interventions strongly conflict with its continued promotion of alcoholic products and have expressed apprehension regarding the alcohol industry’s CSR agenda [12]. In fact, there is little evidence the alcohol industry’s CSR strategies benefit population health or prevent non-communicable diseases [13]. Well-known, evidence-based drink-driving countermeasures do exist, such as a maximum blood alcohol concentration limit of 0.05 g per deciliter for drivers and widespread sobriety checkpoints, but they are rarely used by the alcohol industry [6].

Existing research has extensively covered the alcohol industry’s influence on government, policymaking [4, 9, 13], and marketing [5, 7, 8, 14] to shape law and public perception. However, to our knowledge, we have not found any studies examining the alcohol industry’s influence on road safety NGOs. This is notable, because we know that the alcohol industry seeks to earn credibility by partnering with trusted NGOs to gain legitimacy as a champion of public health [15]. Therefore, it is important to study the depth of the alcohol industry’s involvement with road safety NGOs to understand the impact of this tactic. In light of this research gap, this study seeks to explore the scope of the relationship between the alcohol industry and road safety NGOs globally.

## Methods

A desk review was conducted between July 2020 and March 2021 to meet the study objective. Given that a global census of road safety NGOs does not exist, we conducted a Google search to identify NGOs working on road safety around the world. Keywords were organized around the combination of three main concepts 1) country, 2) road safety, and 3) NGOs (e.g., “India” + “road safety” + “NGO”). Additionally, we reviewed the list of road safety NGOs provided on the WHO website and other international organizations. Through this two-pronged approach, we identified 256 organizations from across the world, representing 92 countries. The local branches of an international NGO were counted as their own entities to understand variation between regions of the world. Sizes of NGOs varied greatly, from small and local, to large and global. Our approach is akin to studies that have also examined large corporate industry influence on public health through involvement with government or NGOs. For example, in their research, Jaichuen et al. [16], for example, also collected data through company websites, domestic, and international social media, government, and private sector organization websites. Further, Mialon et al. [17], recommended a list of sources that could be used to monitor corporate political activity of the food industry, this included industry’s own materials, materials from professional bodies, including websites, and websites of major conferences.

A review of the organization’s website, social media feeds, and secondary searches of the world-wide web (described in further detail below) were conducted to identify ties to the alcohol industry and examine the nature of these relationships. We defined a relationship between road safety NGOs and the alcohol industry as any involvement (e.g., business/financial, personnel-related) with alcohol companies; Social Aspects Public Relations Organizations (SAPROs); “astroturf” groups (i.e., industry-funded front groups that resemble grassroots organizations); or any other entities or persons working on behalf of the alcohol industry. Secondary searches included the name of the NGO being searched, in addition to the main concepts of 1) alcohol industry, 2) drink-driving, and 3) road safety.

Previous reviews relating to drink-driving, road safety, and the alcohol industry were also consulted to find any recent relationships between the alcohol industry and road safety NGOs described in literature. Backwards searching was also utilized, whereby an industry list of the largest alcohol producers and entities was consulted, and research on those entities’ websites was conducted to find mention of affiliated road safety NGOs. Information was obtained in English or translated through Google and Facebook translation services from Arabic, Spanish, Italian, Greek, French, and other languages.

**Fig. 1:**
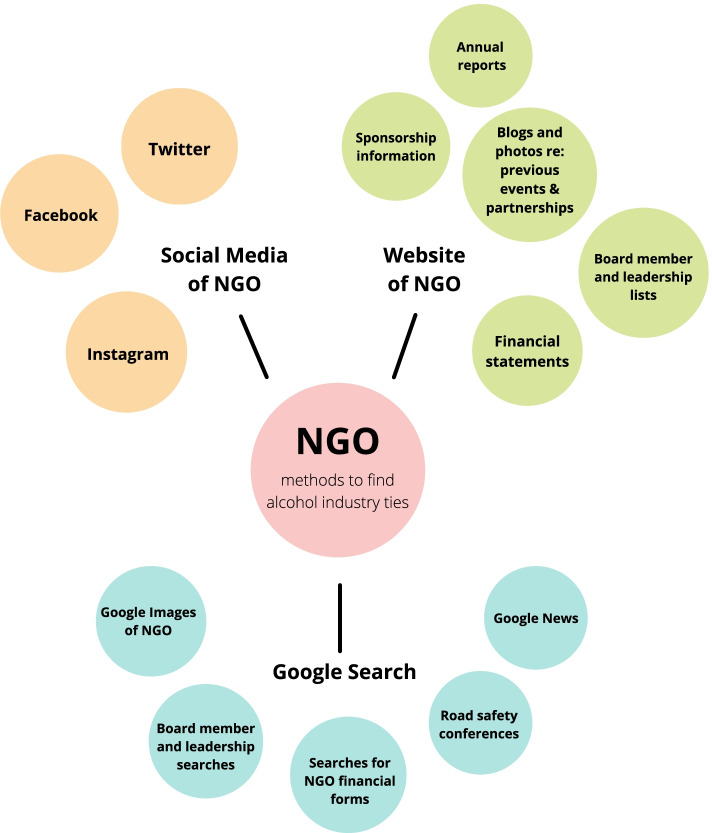
Methods to find road safety NGOs tied to alcohol industry

Information from all 256 NGOs and partnerships were documented in a detailed spreadsheet. Any organizations identified as having direct or indirect ties to the alcohol industry were flagged and researched in further detail through Google searches, financial documents, press releases, social media feeds, websites of the alcohol partners’ websites, and road safety conference materials. All levels of NGO involvement with the alcohol industry were included, from the industry’s local actors to its global corporations and organizations. Once the data were gathered, analysis shifted from examination of relevant content to the aggregation of themes across relationships. These include the types of alcohol actors most involved with road safety NGOs, types of NGOs most involved with the alcohol industry, types of relationships between NGOs and industry actors, types of interventions used, and level of public communication about partnerships (Fig. 1).

### Website of NGO

Websites of the *n* = 256 NGOs were reviewed for signs of a relationship with the alcohol industry in press materials, financial statements, personnel and board member biographies, photographs, blog posts, and other available information. While the majority of NGOs had a functioning website (*n* = 204), others did not (*n* = 52). For the *n* = 204 NGOs with websites, searches were conducted within each NGO website to find a list of sponsors, donors, or partnerships with the alcohol industry. Board members and leadership listed for each NGO were then searched on Google to find any ties to the alcohol industry. Various NGOs (*n* = 112) listed board members and/or management teams on their websites.

Additionally, the websites of the *n* = 204 NGOs were also utilized to find annual reports of each NGO. Commonly, only larger NGOs housed annual reports on the organization’s website (*n* = 32). Recent tax documents (e.g., USA: 990 tax documents) of NGOs were reviewed for potential sponsor and donor information. However, just *n* = 12 NGOs had publicly available tax or financial documents from the last 5 years (2016–2020), while *n* = 244 NGOs did not have tax documents available online. In addition, photographs on *n* = 149 websites were reviewed for any signs of alcohol industry partnership. For example, sponsorship banners from event photographs were used to find an NGO’s alcohol industry partners (*n* = 7). Sponsors were also listed under sponsor or partner sections of some NGO websites (*n* = 5).

### Social media of NGO

In addition to a website search of each NGO, social media searches were undertaken to find ties to the alcohol industry. For all NGOs, Facebook, Twitter, and Instagram from 2016 to 2020 were searched to find any postings that may have involved alcohol partnerships or sponsorships. All *n* = 256 NGOs were present on Facebook, while *n* = 191 were on Twitter, and *n* = 87 were on Instagram. Importantly, all *n* = 52 NGOs without a website had at least one form of presence on a social media platform, which allowed for a gathering of information on each NGO. For *n* = 31 of these 52 NGOs, this was a minimal amount of information – i.e., limited posts and shares (less than five per year), and *n* = 17 were dated by more than 5 years.”

The search functions within Facebook and Twitter were also utilized. For example, keywords like “sponsor,” “partner,” “drink-driving,” “drunk-driving,” “drink,” “nightlife,” “event,” and “substance,” were entered into the search function of each NGO’s Facebook and Twitter to find any posts indicating partnerships with the alcohol industry. Additionally, the “Photos” section of each NGO Facebook profile (*n* = 256) was reviewed from 2016 to 2020 for photographs depicting partnerships with the alcohol industry.

### Google searches

For all NGOs, Google searches were conducted to find alcohol industry sponsors and partners. The organization’s name was used in the search, followed by keywords organized around the three main concepts of 1) alcohol industry, 2) drink-driving, and 3) road safety. For the *n* = 112 NGOs that had personnel lists on their websites, board members and leadership were searched on Google (up to two pages of search results) to find ties to the alcohol industry. Another *n* = 12 NGOs listed names of leadership on their Facebook pages, which were also utilized in personnel Google searches. Additionally, a Google Images search of each NGO was conducted to find images of sponsorship banners or events with the alcohol industry. Similarly, a Google News search was conducted for each NGO coupled with keywords around the concepts of 1) alcohol industry and 2) drink-driving in order to find recent publicity showing potential alcohol industry affiliation.

Furthermore, road safety conferences from 2016 to 2020 (i.e., International Conference on Road Safety, Transport and Road Statistics, Road Safety Performance Index Annual Conference and Award Ceremony, Department for Transport International Road Safety Conference) were researched on Google to uncover any alcohol industry sponsors related to the *n* = 256 NGOs. Moreover, road safety awards (i.e., National Roadway Safety Awards, Prince Michael International Road Safety Awards, Excellence in Road Safety Awards) and alcohol industry corporate social responsibility awards (i.e., CSR Incorporated Awards, The Drinks Business Green Awards, International CSR Excellence Awards) were researched on Google for any NGO and alcohol industry relationships.

## Results

This study showed that out of 256 road safety NGOs, *n* = 11 (4%) were found to have direct ties to the alcohol industry, while *n* = 3 (1%) of 256 road safety NGOs were found to have indirect ties to the alcohol industry. We define indirect ties as any partnerships or sponsorships with entities that may be involved with the alcohol industry (e.g., aluminum can manufacturers, organizations that work with and/or are sponsored by the alcohol industry).

**Fig. 2:**
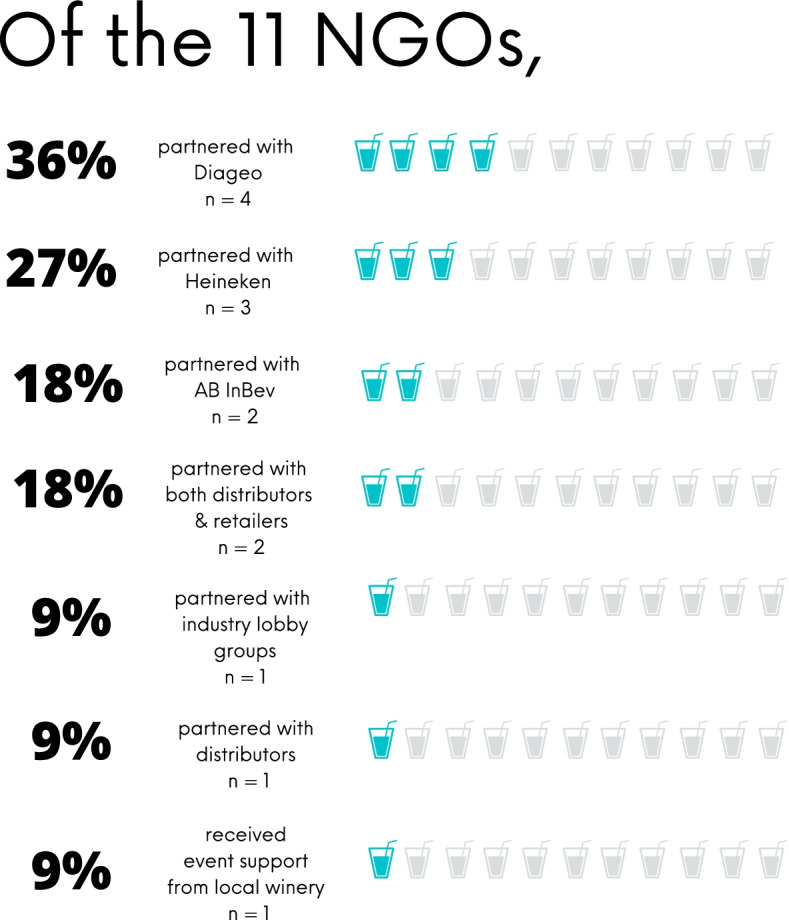
Results of primary research on NGOs directly tied to alcohol industry

Of the *n* = 11 NGOs with direct ties to the alcohol industry, 73% (*n* = 8) were in partnership with transnational alcohol manufacturers such as Diageo, Heineken, and Anheuser-Busch InBev (AB InBev). Of the n = 11 direct partnerships, 36% (*n* = 4) were in partnership with Diageo; 27% (*n* = 3) were in partnership with Heineken; 18% (*n* = 2) were in partnership with AB InBev; 18% (*n* = 2) were in partnership with alcohol retailers and distributors (Woolworths Holdings Limited and Panthera Group); *n* = 1 was in partnership with various industry-funded organizations (Greece’s Responsibility Alliance and the Cyprus Brewers Association) for an annual event; and *n* = 1 received support from a local winery for one event. While *n* = 1 NGO had partnerships with both Diageo, AB InBev, and industry-funded groups, it was more common that NGOs had just one publicly identifiable alcohol industry partner. Interestingly, *n* = 1 of the 11 NGOs was also sponsored by the tobacco industry, specifically a subsidiary of British American Tobacco. This was the only NGO out of 256 reviewed that was found to have direct ties to the tobacco industry (Fig. 2).

None of the *n* = 11 NGOs or alcohol industry partners involved in partnerships publicly detailed or listed all aspects of their relationship. However, NGOs and industry partners for *n* = 3 partnerships posted memos about their partnerships conveying their commitment with language such as, “we will continue to join forces to carry out joint actions to raise public awareness,” “joint actions are planned,” and “[alcohol company] supported activities of the [NGO].” Alternatively, they listed specific efforts together, without clarifying the full extent of the partnership or if more efforts (public-facing and nonpublic-facing) were planned or existed. Various partnerships were not publicly detailed and were found through NGO promotional and marketing materials outlining campaigns and sponsors.

The two most common joint efforts within the *n* = 11 partnerships included mass media campaigns against drink-driving and annual drink-driving awareness events at nightclubs. Mass media campaigns were an aspect of *n* = 4 of the 11 partnerships (36%) between the alcohol industry and road safety NGOs. Commonly, campaigns employed celebrities and athletes and urged the public to sign pacts not to drink and drive. In all *n* = 4 cases, the alcohol manufacturers and retailers included their name in the promotional media materials.

Additionally, annual drink-driving awareness events held in entertainment venues or nightclubs were a common theme in *n* = 3 of the 11 uncovered partnerships (27%) between the alcohol industry and road safety NGOs. These types of events were labeled as harm-reduction interventions using peer-group communication and promoted designated driving, ride-sharing services and/or public transportation. Another annual event series, sponsored by Diageo, was held in nightclubs in the Middle East and included staff training on safe consumption and promotional discount codes for a ride-sharing app.

**Fig. 3:**
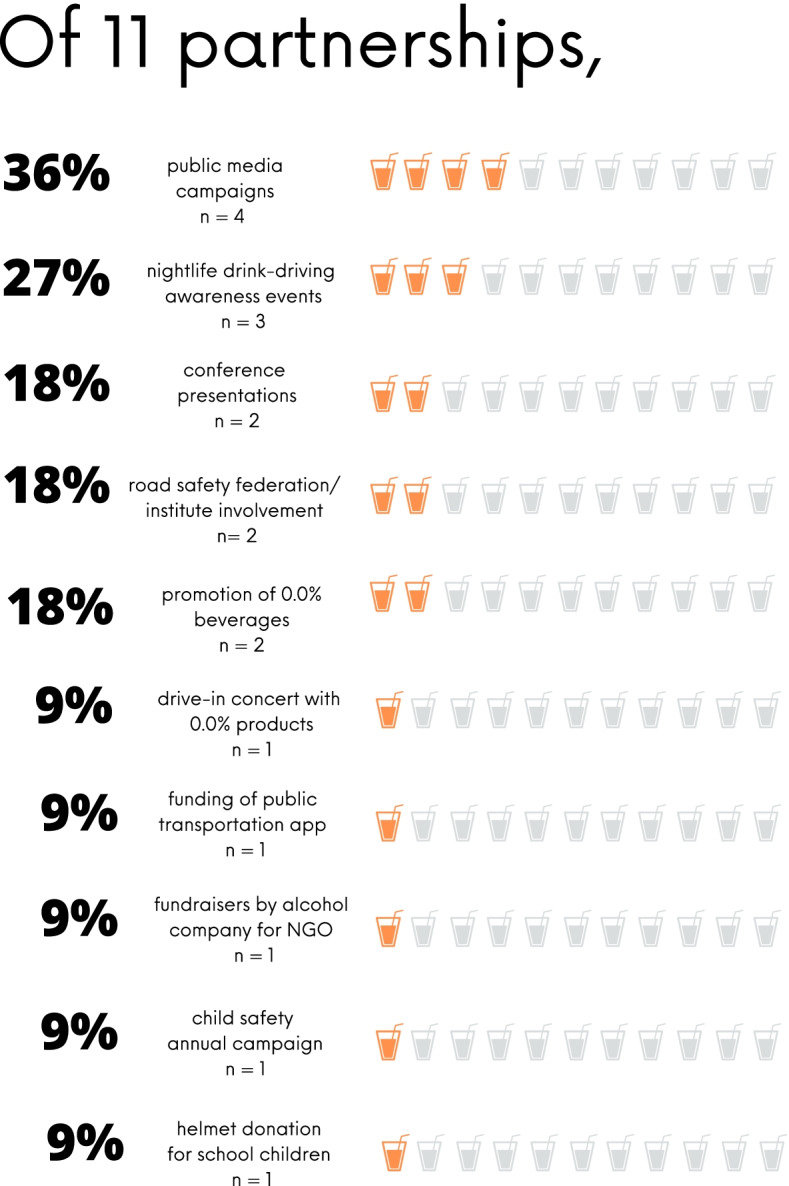
Snapshot of results of primary research on partnerships

**Fig. 4:**
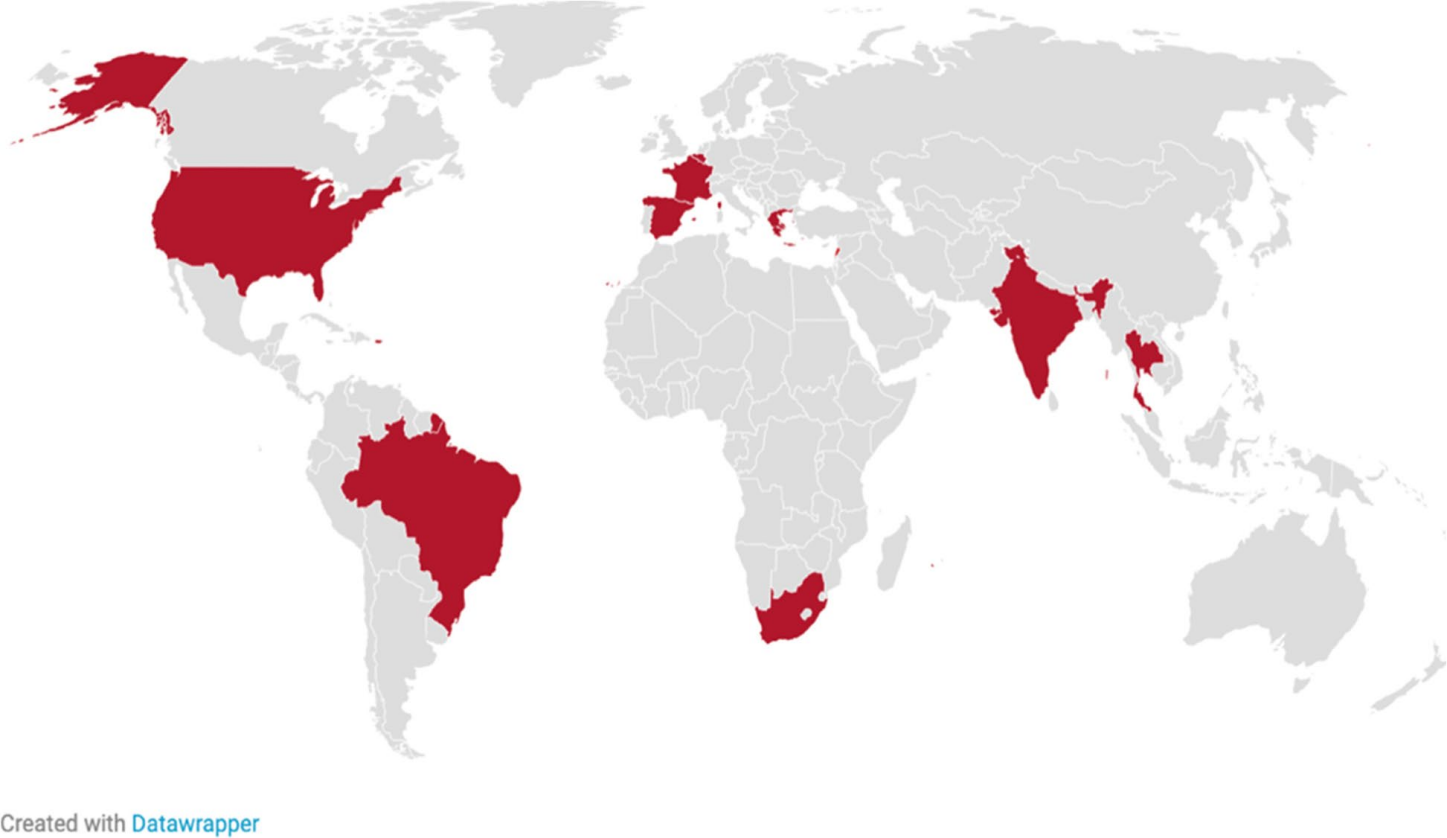
Geographic results of primary research on NGOs directly tied to alcohol industry

The findings of this desk review are consistent with other research documenting the interventions used by the alcohol industry to address drink-driving. Existing literature has found that the majority of these interventions do not follow evidence-based public health recommendations [6, 18]. Such is the case with the majority of interventions we found utilized within the alcohol industry and NGO partnerships: mass media campaigns, educational programs, and educational events with discounted or free ride-sharing [6, 18] (Figs. 3 and 4).

Of the *n* = 11 road safety NGOs found to have direct ties to the alcohol industry, locations of each organization are as follows:

- Europe (*n* = 4)
- Middle East (*n* = 2)
- Asia (*n* = 2)
- Africa (*n* = 1)
- South America (*n* = 1)
- North America (*n* = 1)

In addition to the *n* = 11 NGOs with clear ties to the industry, *n* = 3 NGOs were found to have relationships or interests that may prove to tie them to the alcohol industry. They were found in South America, Europe, and the United States. One of these NGOs was sponsored by an aluminum can manufacturer that frequently partnered with and sold to the beer industry. Another was partners with an entity that had a large, multi-year business deal with Heineken. The third had a supporting partner association with two alcohol industry executives as board members.

## Discussion

These findings demonstrate the alcohol industry’s clear involvement with road safety NGOs around the world. The scope of the involvement is likely to be much more given that transparency is low – not all NGOs publicly showcase their relationship with the industry on their websites and not all have sufficient information online. Additionally, *n* = 1 of the 11 NGOs with direct ties to the alcohol industry also had direct ties to the tobacco industry.

The majority of the interventions used within partnerships between the alcohol industry and road safety NGOs were not consistent with evidence-based best practices. Of the *n* = 11 partnerships examined in our desk review, *n* = 8 partnerships (73%) utilized educational tactics such as public information campaigns, presentations at conferences, and education at nightclub events. Data shows educational interventions like public information campaigns and social marketing are ineffective at decreasing alcohol consumption and alcohol-related harm, while many other programs have not been studied to evaluate success [8, 14, 19]. Additionally, mass media campaigns, a tactic used as a main component of 36% (*n* = 4) of partnerships, are found to be generally ineffective if the emphasis is on limiting alcohol consumption [8, 20]. In fact, educational campaigns and mass media awareness campaigns like the aforementioned have been found to lead to more positive views about alcohol and the alcohol industry among drinkers and nondrinkers alike [18], while bolstering industry sales and public perception of alcohol brands [21, 22]. The findings in this desk review are consistent with existing studies, which show that efforts used to counter drink-driving lack the evidence base necessary to be considered effective [6, 18]. In 2016, Esser et al. [6] found that of 266 global initiatives to decrease drink-driving sponsored by the alcohol industry, only 2 (0.08%) were backed by evidence and recommended by public health experts.

The study also found a lack of transparency regarding disclosure of partnership details (i.e., funding, specifics on the activities encompassed within the partnership) on behalf of both the alcohol industry and road safety NGOs. For all *n* = 11 direct partnerships found within the desk review, *n* = 0 publicly provided comprehensive details of the partnership such as financials or terms and conditions. Given the lack of transparency noticed in the *n* = 11 direct partnerships, it is possible partnerships between the alcohol industry and road safety NGOs may go unnoticed by stakeholders and the public, and if noticed, the scope of the involvement may not be known. The solution to reverse this lack of transparency requires a broad-scale change in the way both road safety NGOs and the alcohol industry publicly report CSR and public health partnerships. Given the public health implications, all stakeholders and the public should be afforded the details and conditions of the partnership and understand the extent to which the alcohol industry is involved in road safety activities of partner NGOs. Without this information, stakeholders and the public are unable to understand possible conflicts of interest.

The findings of this desk review underscore the need for local and national governments to support road safety initiatives so that road safety NGOs are not dependent on funding from health-harming industries, like the alcohol and tobacco industries. Hoe et al. [15] found that road safety NGOs view funding as the key advantage to partnering with the alcohol industry due to minimal funding within the road safety space. Additionally, efforts to improve transparency in the alcohol industry’s relationships with road safety NGOs should be prioritized by governments through legislation, as well as through internal accountability measures within NGOs and the alcohol industry. Both road safety NGOs and the alcohol industry should publicly disclose funders and details of funded activities to increase transparency and allow for accountability.

There is also a need for the road safety community to generate consensus regarding what constitutes acceptable involvement with the alcohol industry. International organizations that work to unite, guide, and support road safety NGOs should enact a policy for member NGOs aimed at increasing awareness of the alcohol industry’s interference within road safety.

While findings demonstrate a strong relationship between the alcohol industry and road safety NGOs, there are some limitations associated with this study. First, assessing alcohol industry involvement solely through online means serves as a limitation when aiming to uncover the full extent of the industry’s involvement with road safety NGOs, as involvement may be kept private or may not be disclosed online. Some NGOs in lower resource settings, for example, may not have the capacity to utilize the internet or do not rely on it for promotion. Moreover, 20% (*n* = 52) of NGOs did not have a functioning website, and as such, more reliance was placed on Google searches and social media for these NGOs. Therefore, less information was available for NGOs without a website. Additionally, we utilized online translation services such as Google and Facebook to assist with translation to English. We are aware that translation services may result in some inaccuracies. Similarly, because our searches only utilized English key terms, there may have been missed information in the online search process. Finally, this desk review examined 256 road safety NGOs globally. It is possible that we did not capture all the road safety NGOs that exist around the world.

Future research could explore the perspectives of road safety NGOs on alcohol industry funding to develop a deeper understanding of the complexities of this topic. This would allow for a greater insight into the reasons various road safety NGOs accept alcohol industry funding, challenges faced when fundraising as a road safety NGO, and potential drawbacks and benefits to accepting alcohol industry support. It would also be insightful to interview the NGOs which actively avoid the alcohol industry.

## Conclusions

The findings of this desk review demonstrate the alcohol industry’s involvement with road safety NGOs around the world. The majority of road safety NGOs in partnership with the alcohol industry were involved with transnational alcohol manufacturers like Diageo, Heineken, and AB InBev, illustrating the influence of global corporations and their corporate responsibility initiatives. While evidence-based drink-driving interventions exist, this study found the alcohol industry partnerships with road safety NGOs largely used interventions without an evidence base. Furthermore, funding details between the alcohol industry and road safety NGOs were not publicly available in any of the partnerships. A clear lack of transparency was found regarding relationships between the alcohol industry and road safety NGOs, meaning the extent of industry involvement in road safety is likely unknown. These findings highlight the need for more government funding of road safety initiatives, more transparency from the alcohol industry and road safety NGOs regarding the alcohol industry’s partnerships with road safety NGOs, and the need for the road safety community to generate consensus regarding involvement of the alcohol industry.

## Data Availability

The datasets used and/or analyzed during the current study are available from the corresponding author on reasonable request.

## Abbreviations

CSR: Corporate Social Responsibility
NGO: Non-Governmental Organization
WHO: World Health Organization
